# Low expression of Ki-67/MIB-1 labeling index in IDH wild type glioblastoma predicts prolonged survival independently by MGMT methylation status

**DOI:** 10.1007/s11060-023-04342-2

**Published:** 2023-05-25

**Authors:** Paolo Tini, Mariya Yavoroska, Maria Antonietta Mazzei, Clelia Miracco, Luigi Pirtoli, Miriam Tomaciello, Francesco Marampon, Giuseppe Minniti

**Affiliations:** 1 Unit of Radiotherapy, Department of Medicine, Surgery and Neurosciences, University of Siena, Siena, Italy; 2 Unit of Diagnostic Imaging, Department of Medicine, Surgery and Neurosciences, University of Siena, Siena, Italy; 3 Unit of Pathological Anatomy, Department of Medicine, Surgery and Neurosciences, University of Siena, Siena, Italy; 4 Center for Biotechnology, College of Science and Technology, Sbarro Institute for Cancer Research and Molecular Medicine, Temple University, Philadelphia, USA; 5 Department of Radiological Sciences, Oncology and Anatomical Pathology, Sapienza University, Rome, Italy; 6 IRCCS Neuromed, Pozzilli, Italy

**Keywords:** Glioblastoma, MIB-1, Ki-67, IDH wild-type

## Abstract

**Purpose:**

The Ki-67/MIB-1 labeling index (LI) is clinically used to differentiate between high and low-grade gliomas, while its prognostic value remains questionable. Glioblastoma (GBM) expressing wild-type isocitrate dehydrogenase IDH^wt^, a relatively common malignant brain tumor in adults, is characterized by a dismal prognosis. Herein, we have retrospectively investigated the prognostic role of Ki-67/MIB-1-LI in a large group of IDH^wt^ GBM.

**Methods:**

One hundred nineteen IDH^wt^ GBM patients treated with surgery followed by Stupp’s protocol in our Institution between January 2016 and December 2021 were selected. A cut-off value for Ki-67/MIB-1-LI was used with minimal p-value based approach.

**Results:**

A multivariate analysis showed that Ki-67/MIB-1-LI expression < 15% significantly correlated with a longer overall survival (OS), independently from the age of the patients, Karnofsky performance status scale, extent of surgery and O^6^-methylguanine (O6-MeG)-DNA methyltransferase promoter methylation status.

**Conclusions:**

Among other studies focused on Ki-67/MIB-1-LI, this is the first observational study showing a positive correlation between OS of IDH^wt^ GBM patients and Ki-67/MIB-1-LI that we propose as a new predictive marker in this subtype of GBM.

## Introduction

Glioblastoma (GBM), the most common malignant central nervous system (CNS) tumor, is characterized by a short overall survival (OS). The standard of treatment for a GBM is surgery, followed by daily radiotherapy (RT) combined with temozolomide (TMZ), then followed by 6 cycles of TMZ [1, 2]. The use of biomarkers predicting prognosis and response to treatment are integrative parts of medical management in GBM patients. In this regard, the methylation status of the gene coding promoter for O6-methylguanine-DNA methyltransferase (MGMT) enzyme has been positively correlated with a prolonged survival in patients treated with TMZ-based therapy [3].

The new classification of tumors of CNS recently introduced, identifies a critical role for the mutation status of the isocitrate dehydrogenase gene (IDH) [4]. The expression of wildtype IDH (IDH^wt^), occurring in ∼90% of all GBM cases, results in a worse prognosis [5], whilst is a weak predictor of long-term survival in GBM patients [6]. Ki-67, a nuclear protein persistently expressed in all phases of the cell cycle, is widely used as a proliferation marker for human tumor cells [7]. MIB-1 is a monoclonal antibody that identifies the Ki-67 protein in paraffin tissue [8]. The Ki-67/MIB-1 labeling index (LI) is one of the immunohistochemical markers used for discriminating between high and low-grade gliomas [9], whilst its use as prognostic factor for the stratification is still discussed [10–20]. Thus, whilst some studies show no association between Ki-67 LI and survival [13, 14, 21], and no predictive value [10, 21], others show a poorer survival rate for lower Ki-67 LI [14, 19], whilst others the opposite [11, 12, 15–18, 20]. Notably, nowadays, no studies have still been performed on the relationship between Ki-67/MIB-1 labeling index and IDH1-WT status in GBM.

In the present study, we have investigated the power of Ki-67/MIB-1 expression as prognosticator in a large and homogenous group of patients suffering from IDH wild-type Glioblastoma (IDH^wt^ GBM) prognostic impact of the Ki-67/MIB-1 labeling index.

## Patients and methods

### Patient characteristics

Between February 2016 and July 2021, 183 consecutive patients with GBM were treated at University Hospital of Siena, Italy. The main clinical data (extent of surgery, clinical examination, blood counts and chemistry, Karnofsky Performance Status – KPS) were registered in all patients. All GBMs were surgically removed and characterized for the MGMT methylation-, IDH1 mutation status, and Ki-67/MIB-1-LI score. One hundred and nineteen patients, characterized for MGMT status and IDH-wild type and MIB-1/Ki 67 labeling index are selected for the present analysis. Characteristics of patients are listed in Table 1. All patients received RT plus concomitant daily TMZ, followed by adjuvant TMZ. RT started within 6 weeks of surgery and consisted of fractionated focal irradiation, at the dose of 60/59.4 Gy in 30/33 fractions of 2/1.8 Gy each. Concomitant chemotherapy consisted of TMZ at the dose of 75 mg/m^2^, given 7 days per week from the first day of RT. Adjuvant TMZ was started 4 weeks after the end of RT and delivered for 5 days every 28 days up to 12 cycles. The dose was 150 mg/m^2^ for the first cycle and was increased to 200 mg/m^2^ from the second cycle. The dose was reduced or suspended in patients with disease progression or toxicity. MRI was repeated before RT, before the first cycle of adjuvant TMZ, and thereafter every 8 weeks or as appropriate according to neurological status. Neuroradiographic response was assessed by RANO criteria [22]. Tumor progression was defined by an increase in tumor size more than 25% or by the presence of a new lesion on imaging. Radiological progression had to be confirmed at two different MRI evaluations (at least 2 months apart). In patients with tumor progression, the recurrence was recorded at the time of the first MRI showing progression.

**Table 1:** Patient’s demographics and survival parameters (univariate and multivariate analysis)

Parameters		Number of patients	Median survival	Univariate analysis (p-value)	Multivariate analysis (p-value) - OR (CI 95%)
Age	< 55 y	20	30 months	p = 0.043	
	> 55 y	99	11 months		
Karnofsky performance status	100–80	103	14 months	p = 0.000	3.46; 95% CI: 1.83–6.54; p = 0.000
	< 70	16	6 months		
Extent of surgery	Gross total	25	23 months	p = 0.048	2.04; 95% CI: 1.08–3.84; p = 0.001
	Sub-total–biopsy	94	11 months		
Radiotherapy total dose	< 59.4/60 Gy	27	8 months	p = 0.001	
	59.4–60 Gy	70	15 months	p = ns	
	> 60 Gy	22	15 months		
Radiological treatment response (6 months)	Complete	23	60 months	p = 0.000	
	Partial	25	21 months		
	Stable	20	17 months		
	Progression	51	8 months		
MGMT promoter status	Methylated	51	25 months	p = 0.000	2,83; 95% CI: 1,20–3,32 p = 0.023
	Unmethylated	68	8 months		
Ki-67/ Mib-1% class	Mib-1 < 15%	17	40 months	p = 0.005	3,85; 95% CI: 1,84–4.43; p = 0.001
	Mib-1 > 15%	102	11 months		

### Treatment planning and treatment parameters

Radiation treatment planning was performed with the Varian Eclipse Treatment Planning System. In each patient, the treatment volume was delineated using post-contrast thin-slice (1-mm) gadolinium-enhanced T1-weighted and T2-weighted MRI axial sequences fused with planning computed tomography (CT) scans of 1.2 mm acquired throughout the entire cranium. The gross tumor volume (GTV) encompassed the resection cavity and any residual tumor as seen on a contrast enhancing T1 postoperative MRI. Delineation of clinical target volume (CTV), considered to contain the microscopic disease, was carried out by adding a margin of 2 cm to the GTV (standard-CTV plan). The CTV margins were reduced to 1–3 mm around natural barriers to tumor growth (the skull, ventricles, falx, etc.), as well to allow sparing of the optic nerve/chiasm, if necessary. The CTVs were expanded by 5 mm to create the planning target volumes (PTV) to compensate for variability in treatment setup and patient motion. The prescribed dose was normalized to 100% at the isocenter and 95% isodose surface covered the PTV as the minimum dose (ICRU Report 50). Treatment was given using a Tomotherapy machine. Normal tissue was contoured to include cerebral hemispheres, hippocampi, brainstem, optic nerves, and chiasm, eyes, and cerebellum. Maximum dose was 55 Gy to the eyes, optic nerve, or chiasm, and 54 Gy to the brainstem. The treatment was performed with the Raystation Planning System. The local Institutional Review Boards approved the study.

### MGMT status and MIB-1/Ki67 evaluation

We assessed the MGMT gene promoter methylation status using a methylation-specific Polymerase Chain Reaction (PCR), as previously reported [23]. Briefly, genomic DNA was extracted from paraffin-embedded tumor sections and treated with sodium bisulfite using the EZ DNA Methylation-Gold kit (HISS Diagnostics, GmbH, Freiburg, Germany). Primer sequences were used to detect methylated and unmethylated MGMT promoter sequences. PCR products were separated on 2% agarose gel. A glioma cell line with a completely methylated MGMT promoter, and peripheral blood mononucleated cells, served as positive and negative control samples, respectively. A methylation percentage of 5% was used as a cut-off value: samples with methylation < 5% and > 5% were classified as unmethylated (Unmet MGMT) and methylated (MethMGMT), respectively.

Evaluation of MIB-1 Expression: Protein expression was determined by neuropathological evaluation of biopsy or resection tissue. Immunohistochemistry was performed. In brief, heat-induced epitope retrieval was performed with either citrate or ethylenediaminetetraacetic acid (EDTA) according to the manufacturer’s protocol of the respective primary antibody. Sections were incubated for 1 h with the following primary antibodies anti-Ki-67/MiB-1 (1:200; Dako M7240, Agilent Technologies, Inc., Santa Clara, CA, USA). Sections were washed and incubated with post-block solution and horseradish peroxidase (HRP) polymer reagent according to the manufacturer’s protocol of the ZytoChem-Plus HRP Polymer Kit (Zytomed Systems GmbH, Berlin, Germany). Ki-67 Labeling Index/MIB-1 demonstrates the percentage of immunoreactive tumor cells from all tumor cells.

### Statistical analysis

For data collection and analysis, we used IBM® SPSS® Statistics (version 21; IBM Corp., Armonk, NY, USA). The prevalence of investigated variables as well as the calculation of means and standard deviations was obtained by descriptive statistics. Comparison between nominal variables have been made with Chi2 test. Continuous variable correlations have been investigated with Pearson’s Bivariate correlation. Threshold of statistical significance was considered p < 0.05. Overall survival (OS) and progression-free survival (PFS) in patients with recurrent or progressive tumors were estimated using the Kaplan–Meier method calculated from the time of radiation treatment to the date of death from any cause. All tests with p < 0.05 were then included in univariate analysis (log-rank test) for comparison of survival probability. Following this, all tests with p < 0.1 were included in multivariate analysis using a Cox proportional hazards model to analyze possible dependencies. Lastly, tests with p < 0.05 in multivariate analysis were considered significant. The assessment of Ki67/MIB-1-LI as survival prognosticator was performed using software X-TILE that allows to define the best cut-off point for biomarkers with minimal p-value [24]. This is an outcome-based cut-point optimization approach that illustrates the presence of substantial tumor subpopulations and shows the robustness of the relationship between a biomarker and outcome by construction of a two-dimensional projection of every possible subpopulation.

## Results

**Fig. 1:**
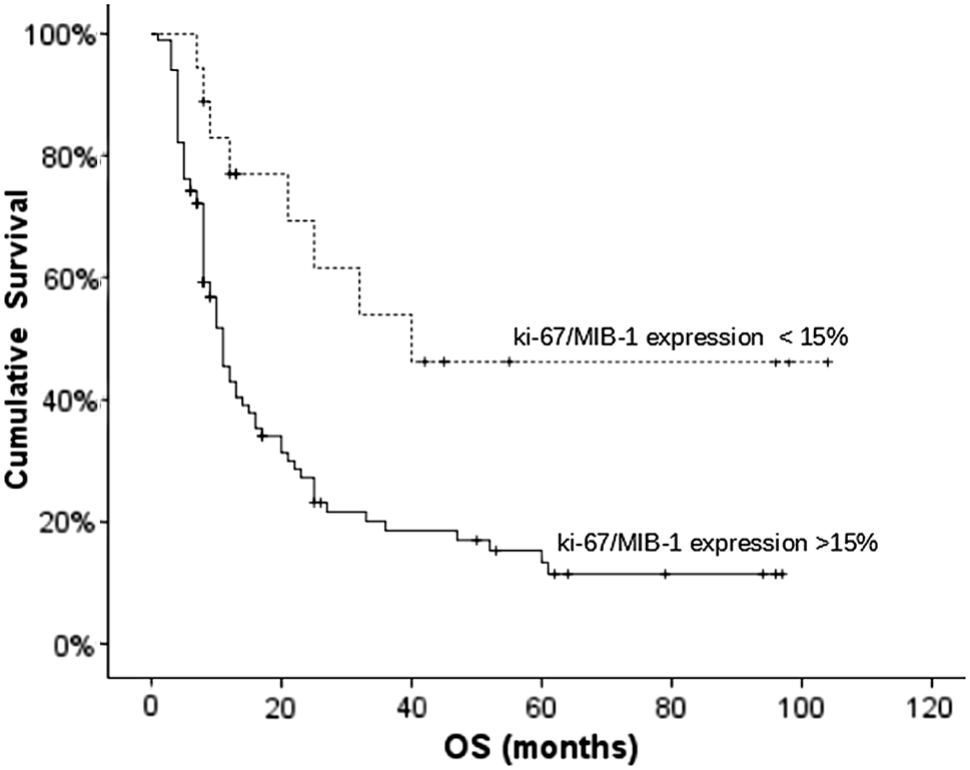
Kaplan Meier Survival curves according Mib-1 index (cut-off: 15%) in all patients selected for the present analysis (119 pts, p = 0,005). Abbreviations: *OS*: averall survival

In the selected population, after a median follow-up time of 18 months [range 2–76 months], the median OS was 12 months, with 78.2% and 48.5% survival rates at 6 and 12 months, respectively. Median PFS was 7 months, with 55% and 33.6% survival rates at 6 and 12 months, respectively. Patients characteristics, age, KPS, extent of surgery, RT dose, Radiological Response, MGMT status and Ki67/MIB-1-LI and the corresponding OS data, are reported in Table 1. Regarding the MGMT promoter status, it was unmethylated in 68 cases (57.1%) and methylated in 51 (42.9%). After the survival univariate analysis (Table 1), significant factors for OS were: KPS, extent of surgical resection, RT dose, age, MGMT status, response to treatment. Moreover, we identified a most-significative cut-off value for MIB-1 of 15% of expression with a survival value (p = 0.005). The patients with a Ki67/MIB-1-LI value < 15% were 17 and had median survival 40 months, 102 patients with Ki67/MIB-1-LI value > 15% had a median survival 11 months (Fig. 1). Distribution and correlation analysis between the MIB-1 expression and other prognostic parameters showed that the MIB-1 expression level is not significantly associated with other prognostic factors: such as KPS, extent of surgery, MGMT, age and Radiological Response. On the other hand, combined MGMT status is strongly correlated to the radiological response to treatment (p = 0.000).

Multivariate analysis (Cox regression analysis) showed that KPS (HR: 3.46; 95% CI: 1.83–6.54; p = 0.000), extent of surgery (HR: 2.04; 95% CI: 1.08–3.84; p = 0.001), MGMT (HR: 2.83; 95% CI: 1.20—3.32 p = 0.023) and Ki67/MIB-1-LI status (HR: 3.85; 95% CI: 1.84—4.43; p = 0.001) were independent prognostic factors.

**Fig. 2:**
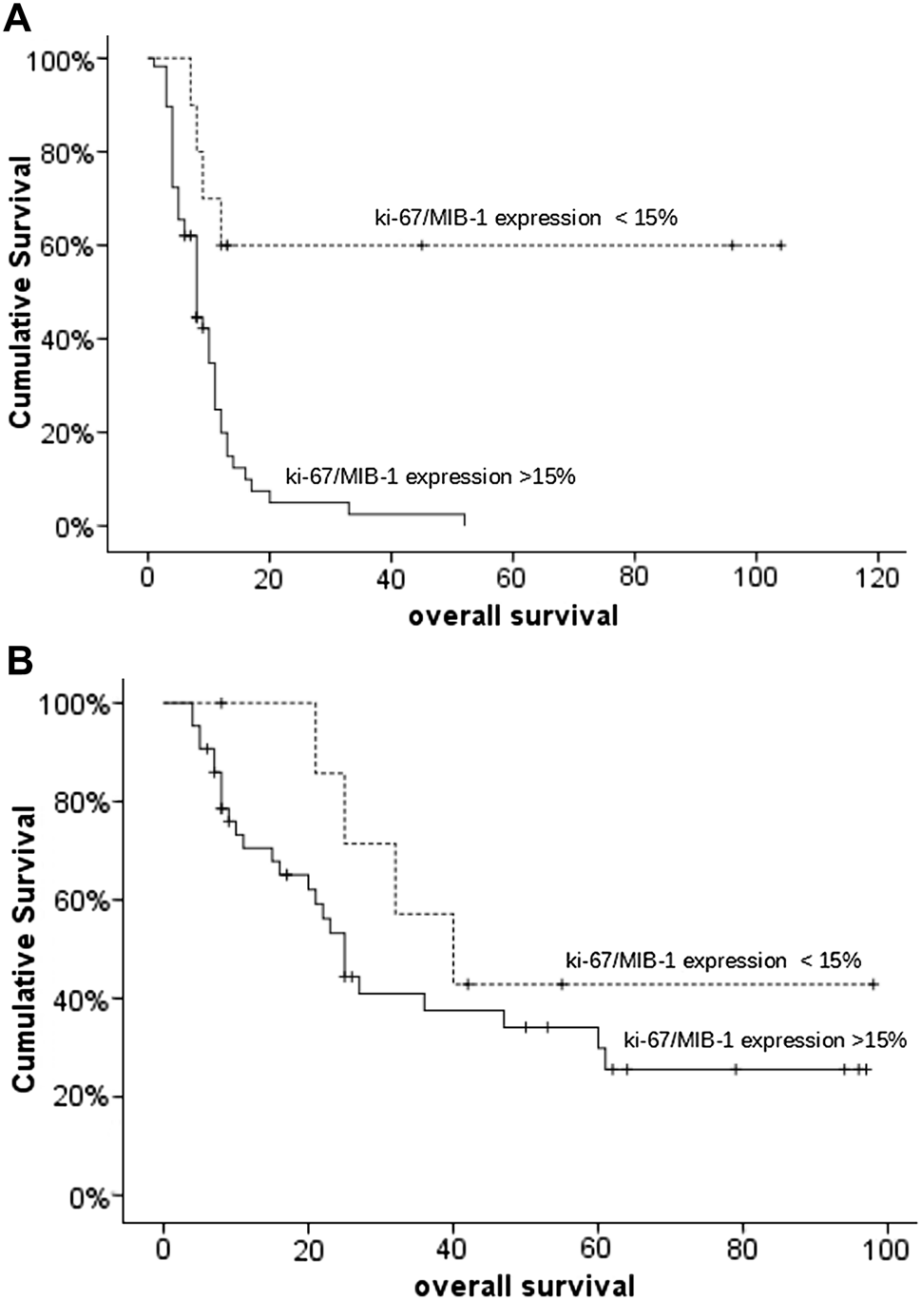
Kaplan Meier Survival curves according to the Mib-1 index (cut-off: 15%) and MGMT status. **A** Methylated MGMT patients (51 patients; p = 0,03). **B** Unmethylated MGMT patients (68 patients, p = 0,02)

Interestingly, low Ki67/MIB-1-LI values were independently associated with survival, identifying long survival patients in the methylated and unmethylated patients. Indeed, the Methylated MGMT-MIB-1 < 15% group was associated with the longest OS (8 patients; median OS 41 months); Methylated MGMT-MIB-1 > 15% group (43 patients;) has a median OS 25 months (p = 0,003); in the Unmethylated MGMT-MIB-1 > 15% group (59 patients; median OS 8 months; in the Unmethylated MGMT-MIB1 < 15% group containing 9 patients median OS is not reached (Fig. 2A and B).

## Discussion

Concurrent and sequential TMZ with RT, after complete surgical removal is the standard treatment for newly diagnosed GBMs. The overall expected 5-year survival rate for GBM patients is < 5% [25], and several data suggest that survival depends on a combination of intrinsic patient characteristics and genetic mutations.

In neuro-oncology, the Ki67/MIB-1-LI is widely used [21], with the expression of Ki 67/Mib-1 ≥ 10% e IDH^wt^ strongly suggestive of GBM diagnosis. However, the prognostic role of Ki-67/MIB-1-LI remains largely debated, with large discrepancies [10–19], potentially depending on the inter- and intra-observer variability [26–28], and lack of standardization in the immunostaining procedure [29]. Furthermore, the prognostic role of Ki-67/MIB-1-LI has been often investigated considering other prognostic factors, rather than directly analyzing the correlation with OS [20], and only few papers approached the association considering histological heterogeneity [30, 31]. Therefore, nowadays, it is not yet a cut-off point for Ki67/MIB-1-LI capable of having a potential prognostic effect [19, 31, 32]. In the present work we demonstrate a prognostic significance for Ki67/MIB-1-LI in IDH^wt^ GBM patients, with a cut off level 15%. The incidence of IDH^wt^ GBMs with a Ki-67/Mib-1-LI lower than 15% are quite rare and is found only in 17 patients among 119 patients but is strongly and independently associated with a long survival with a median survival of 40 months. Thus, our evidence confirms what has already been previously described [19], with the important difference that our evidence indicates a survival of 40 months and not 18 months previously indicated [19].

Notably, when combined with MGMT status, Ki67/MIB-1-LI correlates with a higher OS of IDH^wt^ patients, independently from MGMT promoter status. The correlation analysis didn’t clarify the modality in which low Ki67/MIB-1-LI provides a better prognosis, indeed it wasn’t correlated with any analyzed prognostic factors, nor to the radiological treatment response. In consideration of a homogeneity of treatment for the patients selected in the work, we believe that an explanatory hypothesis could be that the parameter ki/67/MIB-1 is not only a prognostic factor but also a predictive factor of response to radio-chemotherapy treatment.

## Conclusions

Ki67/MIB-1-LI used with a cut-off value of 15% seems to be very interesting as a prognostic-related index in IGH^wt^ patients, identifying those candidates to have a higher OS, independently by MGMT status. The retrospective analysis setting, the mono-centric data and, particularly, the uneven group sizes are the main limitations of the present work. Our study results present an interesting finding that warrants further investigation, perhaps in the first instance through larger retrospective studies involving multiple cancer treatment and pathology centers. More data should be collected in a prospective and multi centric setting to overcome the discrepancy of Mib-1 expression assessment due to inter-and intra-observer variability.
